# Spontaneous Pneumothorax in a Young Female With Lymphangioleiomyomatosis

**DOI:** 10.7759/cureus.45413

**Published:** 2023-09-17

**Authors:** Osato Ukponmwan, Asher Gorantla, Krunal H Patel, Elmer Gabutan, Li Zhonghua, Samy I McFarlane

**Affiliations:** 1 Internal Medicine, State University of New York Downstate Health Sciences University, Brooklyn, USA; 2 Pathology, State University of New York Downstate Health Sciences University, Brooklyn, USA; 3 Pathology, Kings County Hospital Center, New York, USA

**Keywords:** vegf-d, hydropneumothorax, pneumothorax, lymphangioleiomyomatosis

## Abstract

Lymphangioleiomyomatosis (LAM) is a rare disorder of abnormal proliferation of smooth muscle-like cells which results in the formation of thin-walled cysts and progressive lung destruction. It commonly presents with progressive dyspnea that is often associated with a history of pneumothorax or chylothorax particularly among females of reproductive age.

In this report, we present a case of hydropneumothorax as the initial presentation of LAM in a 33-year-old woman, a rather rare presentation. We also discuss the pathogenetic mechanisms, the diagnosis, and treatment strategies using mTOR inhibitors like sirolimus.

## Introduction

Lymphangioleiomyomatosis (LAM) is a rare disorder of abnormal proliferation of smooth muscle-like cells which results in the formation of thin-walled cysts and progressive lung destruction [1]. It is a multi-organ disease that primarily affects females of reproductive age and is generally diagnosed incidentally. This disease is extremely rare; to date, it is documented through case reports. LAM typically presents as diffuse thin-walled cysts surrounded by normal lung parenchyma on CT imaging. A high vascular endothelial growth factor-D (VEGF-D) value greater than 800pg/ml is diagnostic for LAM in patients with typical CT findings. Alternatively, a lung biopsy may demonstrate the proliferation of atypical smooth muscle-like cells, normal lung architecture distorted by multiple small cysts, and HMB-45 staining positivity. This report presents new onset LAM in a young female with hydropneumothorax.

This article was previously presented as a meeting abstract at the 2022 SUNY Downstate Research Day on May 31, 2022.

## Case presentation

**Figure 1:**
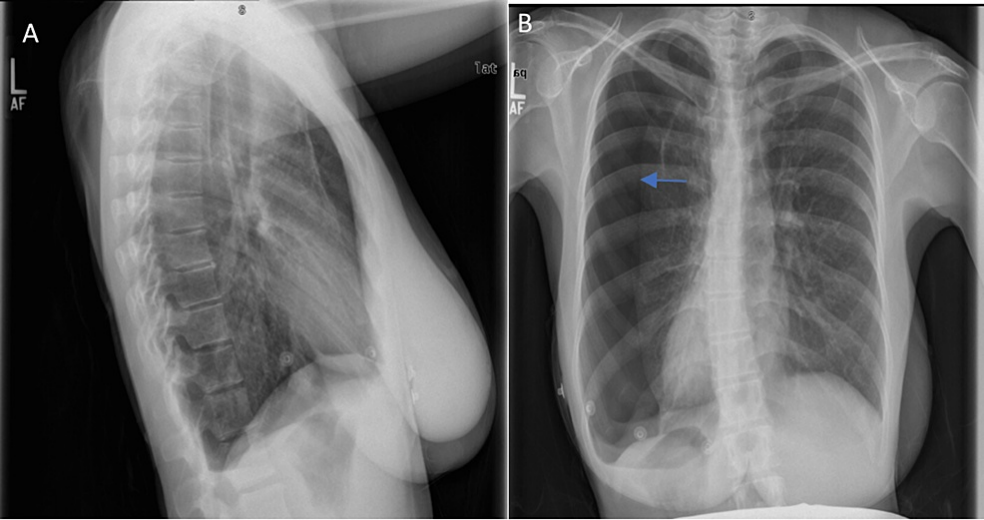
Initial Chest X-ray from the Emergency Room Emergency Room Chest X-ray positive for large left-sided hydropneumothorax (arrow), with mild mediastinal shift to the right. Figure 1A shows a lateral X-ray and Figure 1B is a posterior-anterior X-ray. A large left-sided hydropneumothorax is highlighted by the arrow.

**Figure 2:**
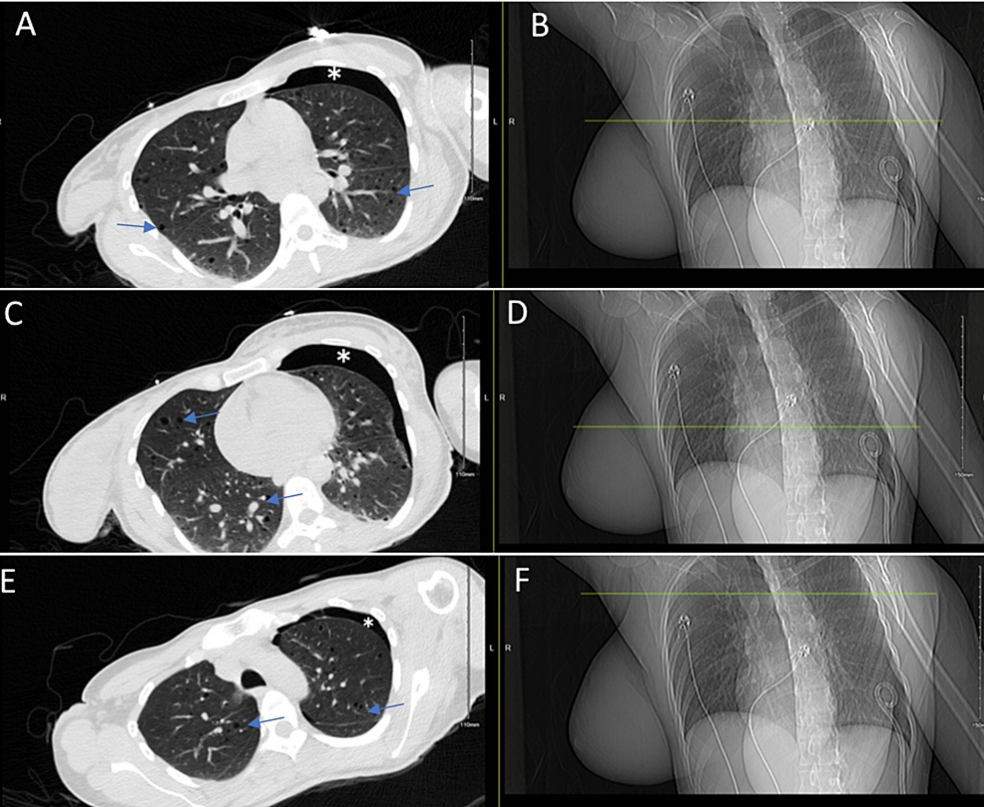
CT and corresponding Chest X-ray images CT imaging with corresponding Chest X-ray significant for left-sided pneumothorax, thin-walled cysts (arrows) diffusely throughout lungs with ground glass background suspicious for lymphangioleiomyomatosis. Area of pneumothorax highlighted by Asterisk (*). Figure 2A and Figure 2B demonstrate cysts and pneumothorax in the middle lung zones. Figure 2C and Figure 2D demonstrate cysts and pneumothorax in lower lung zones. Figure 2E and Figure 2F highlight cysts and pneumothorax in upper lung zones.

**Figure 3:**
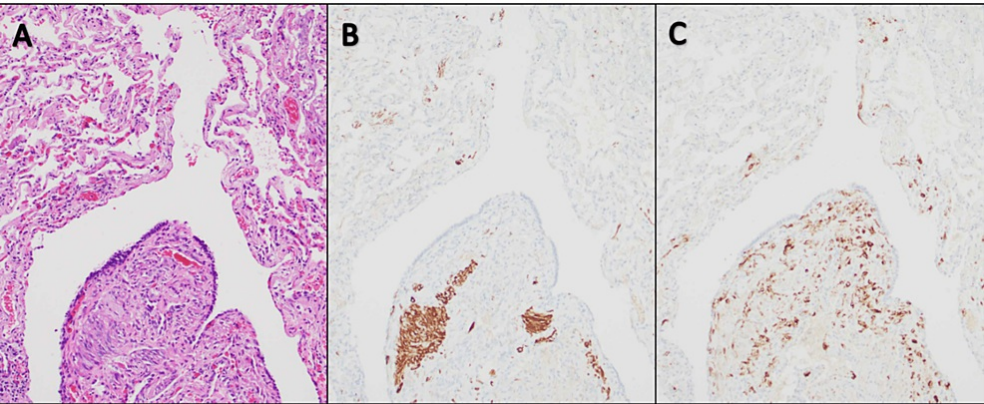
Lung Resection Lung Pathology Specimen Lymphangioleiomyomatosis was confirmed by lung wedge resection. Wedge resection showed pulmonary parenchyma with scattered thin-walled cystic air spaces with patchy, disordered, scattered to nodular proliferation of bland spindle cells and scattered cuboidal epithelioid cells in the cystic wall (A, H&E, x100). These spindle cells are positive for smooth muscle markers smooth muscle actin (B, Immunohistochemistry, x100) and desmin (data not shown), and cuboidal epithelioid cells are positive for HMB-45 (C, Immunohistochemistry, x100).

A 33-year-old woman with a noncontributory past medical history presented to the emergency department with sudden onset pleuritic chest pain that occurred while pushing her child’s stroller. The pain improved with rest but recurred more severely while at work prompting her to call emergency medical services. The patient does not report similar episodes, recent trauma, or lung disease. In the emergency department, she appeared uncomfortable, with decreased left-sided breath sounds. Chest pain was worse with the movement of her left arm. ECG showed normal sinus rhythm without previous ECGs for comparison. Routine biochemical profiles including complete blood count, comprehensive metabolic panel, activated partial thromboplastin time, and D-Dimer were within normal limits and COVID-19 testing was negative. Chest X-ray (Figure 1) was significant for large left-sided hydropneumothorax with a mild mediastinal shift to the right. A pigtail catheter was placed. CT imaging (Figure 2) was performed to investigate secondary causes of pneumothorax, which showed thin-walled cysts diffusely throughout the lungs with a ground glass background suspicious for LAM. Left video-assisted thoracoscopy (VATS) with left lung wedge resection was performed by Cardiothoracic surgery, confirming the diagnosis of LAM (Figure 3). The patient was discharged with a pulmonary follow-up and initiated treatment with Sirolimus.

## Discussion

Lymphangioleiomyomatosis (LAM) is a diffuse cystic lung disease that predominantly affects young females. There are two main types of LAM - sporadic and associated with tuberous sclerosis (TSC). Clinically, it is characterized by cystic lung disease, renal angiomyolipomas (AMLs), and lymphatic complications [2,3]. The clinical features result from progressive cystic destruction of the lungs and the accumulation of LAM cells within the lungs and axial lymphatics. The disease is rare (termed sporadic LAM) with a prevalence of ∼1 in 1,000,000 people, with 40% of patients with TSC affected [4].

LAM associated with the tuberous sclerosis complex involves loss of function mutations in the TSC1 or TSC2 genes which encode hamartin and tuberin [5]. As a result, the rapamycin (mTOR) signaling pathway is activated, leading to inappropriate LAM growth, proliferation, and metastatic spread of LAM cells [6]. In addition, LAM cells also express lymphangiogenic growth factors (vascular endothelial growth factor (VEGF)-C and VEGF-D), which are involved in the metastatic spread of LAM cells. LAM cells also induce the breakdown of the extracellular matrix by matrix metalloproteinases (MMPs) possibly contributing to cyst formation as they have been detected in tissue in cystic areas in the lung.

AMLs occur in 30% of patients with sporadic LAM and 80% of patients with LAM associated with tuberous sclerosis. In S-LAM, AMLs are usually unilateral and asymptomatic. A minor percentage of patients with LAM present with hemorrhage secondary to an AML [7]. In patients with TSC and those with lymphatic abnormalities or AMLs, careful evaluation to exclude LAM is needed. Blood work includes screening for α1-antitrypsin, connective tissue disease screen (anti-Ro/La, anti-cyclic citrullinated peptide, rheumatoid factor, and antinuclear antibody), and lymphoproliferative disorders.

Presenting features usually involve the respiratory system, commonly with progressive dyspnea, chylous pleural effusions, and pneumothorax, as was seen in our patient. Dyspnea is by far the most common complaint in these patients and develops due to airflow obstruction and replacement of lung parenchyma by cysts. Approximately two-thirds of patients develop pneumothorax over their clinical course, which is a cause of significant morbidity.

In a patient presenting with clinical features of LAM, high-resolution computed tomography is the modality of choice to visualize pulmonary cysts. By current guidelines put forth by the American Thoracic Society, a definitive diagnosis of LAM can be made based on the presence of multiple characteristic cysts on lung HRCT and any of the following: renal AML, thoracic or abdominal chylous effusion, lymphangioleiomyomas or lymph nodes involved in LAM, TSC, and elevated VEGF-D (>800 pg/mL) [8].

While a common presentation of LAM includes progressive dyspnea that is often associated with a history of pneumothorax or chylothorax, our patient presented with hydropneumothorax as her initial presentation. Up to 70% of patients with LAM will develop pneumothorax during their lifetime and there is a very high risk of recurrence [9]. Although constituting a small fraction of pneumothoraces, LAM should be suspected in women of reproductive age presenting with pneumothorax on chest X-ray without otherwise plausible etiology. A CT chest with scattered thin-walled rounded cysts increases suspicion of LAM. Diagnosis can be confirmed by lung biopsy.

European Respiratory Society LAM guidelines and the American Thoracic Society/Japanese Thoracic Society guidelines [8] recommend chemical pleurodesis and surgical intervention, respectively, for the first pneumothorax. Treatment revolves around the use of mammalian target of rapamycin (mTOR) inhibitors like sirolimus which are effective at stabilizing lung function and reducing the size of effusions, lymph angioleiomyomas, angiomyolipomas, and VEGF-D levels [5]. VEGF-D levels <800 pg/mL and early sirolimus treatment are associated with delayed decline in FEV1 and increased eight-year cumulative survival rates [10].

## Conclusions

Currently, no single clinical or serological factor has been shown to predict prognosis. Physicians should be aware of such a rare condition, particularly in an at-risk population presenting with pneumothorax, since treatment can slow the progression of the disease. Continued advances in understanding the molecular basis of LAM will lead to improved therapeutic targets and the development of more robust prognostic indicators. Our patient had VEGF levels < 800 and was started on sirolimus early which is associated with increased survivorship.
